# Development and external validation of DISPAIR fistula risk score for clinically relevant postoperative pancreatic fistula risk after distal pancreatectomy

**DOI:** 10.1093/bjs/znac266

**Published:** 2022-08-19

**Authors:** Akseli Bonsdorff, Poya Ghorbani, Ilkka Helanterä, Timo Tarvainen, Tea Kontio, Hanna Belfrage, Jukka Sirén, Arto Kokkola, Ernesto Sparrelid, Ville Sallinen

**Affiliations:** Gastroenterological Surgery, Helsinki University Hospital and University of Helsinki, Helsinki, Finland; Division of Surgery, Department of Clinical Science, Intervention, and Technology, Karolinska Institutet, Stockholm, Sweden; Transplantation and Liver Surgery, Helsinki University Hospital and University of Helsinki, Helsinki, Finland; Gastroenterological Surgery, Helsinki University Hospital and University of Helsinki, Helsinki, Finland; Transplantation and Liver Surgery, Helsinki University Hospital and University of Helsinki, Helsinki, Finland; Gastroenterological Surgery, Helsinki University Hospital and University of Helsinki, Helsinki, Finland; Gastroenterological Surgery, Helsinki University Hospital and University of Helsinki, Helsinki, Finland; Transplantation and Liver Surgery, Helsinki University Hospital and University of Helsinki, Helsinki, Finland; Gastroenterological Surgery, Helsinki University Hospital and University of Helsinki, Helsinki, Finland; Division of Surgery, Department of Clinical Science, Intervention, and Technology, Karolinska Institutet, Stockholm, Sweden; Gastroenterological Surgery, Helsinki University Hospital and University of Helsinki, Helsinki, Finland; Transplantation and Liver Surgery, Helsinki University Hospital and University of Helsinki, Helsinki, Finland

## Abstract

**Background:**

Highly utilized risk scores for clinically relevant postoperative pancreatic fistula (CR-POPF) have guided clinical decision-making in pancreatoduodenectomy. However, none has been successfully developed for distal pancreatectomy. This study aimed to develop and validate a new fistula risk score for distal pancreatectomy.

**Methods:**

Patients undergoing distal pancreatectomy at Helsinki University Hospital, Finland from 2013 to 2021, and at Karolinska University Hospital, Sweden, from 2010 to 2020, were included retrospectively. The outcome was CR-POPF, according to the 2016 International Study Group of Pancreatic Surgery definition. Preoperative clinical demographics and radiological parameters such as pancreatic thickness and duct diameter were measured. A logistic regression model was developed, internally validated with bootstrapping, and the performance assessed in an external validation cohort.

**Results:**

Of 668 patients from Helsinki (266) and Stockholm (402), 173 (25.9 per cent) developed CR-POPF. The final model consisted of three variables assessed before surgery: transection site (neck *versus* body/tail), pancreatic thickness at transection site, and diabetes. The model had an area under the receiver operating characteristic curve (AUROC) of 0.904 (95 per cent c.i. 0.855 to 0.949) after internal validation, and 0.798 (0.748 to 0.848) after external validation. The calibration slope and intercept on external validation were 0.719 and 0.192 respectively. Four risk groups were defined in the validation cohort for clinical applicability: low (below 5 per cent), moderate (at least 5 but below 30 per cent), high (at least 30 but below 75 per cent), and extreme (75 per cent or more). The incidences in these groups were 8.7 per cent (11 of 126), 22.0 per cent (36 of 164), 63 per cent (57 of 91), and 81 per cent (17 of 21) respectively.

**Conclusion:**

The DISPAIR score after distal pancreatectomy may guide decision-making and allow a risk-adjusted outcome comparison for CR-POPF.

## Teaser

This study describes the development and external validation of the DISPAIR score, a preoperative clinical prediction model to estimate the risk of postoperative pancreatic fistula after distal pancreatectomy. It is based on three variables measured before operation: pancreatic thickness, transection site, and history of diabetes. On external validation, it showed satisfactory discrimination (area under the curve 0.80) and calibration (slope 0.719, intercept 0.192) for predicting pancreatic fistula.

## Introduction

Clinically relevant postoperative pancreatic fistula (CR-POPF) accounts for most of the morbidity after distal pancreatectomy (DP). Regardless of CR-POPF mitigation strategies after DP, such as pancreatic stump coverage with an autologous tissue patch, administration of perioperative somatostatin analogues, and use of intra-abdominal drainage, its incidence remains higher than that after pancreatoduodenectomy^1–4^.

Preoperative risk estimation could advance efforts to prevent CR-POPF after DP. The development and application of the fistula risk score^5^ for pancreatoduodenectomy has made risk-adjusted comparisons of patient outcomes possible and guided the use of mitigation strategies. However, no corresponding model has been successfully developed for DP. Ecker *et al*.^6^ conducted a study with over 2000 patients, but they were not able to reliably predict CR-POPF after DP. In their analyses, young age, high BMI, hypoalbuminaemia, absence of epidural anaesthesia, non-malignant pathology, concomitant splenectomy, and vascular resection were independent risk factors; however, the proposed prognostic model showed poor discrimination. Moreover, no pancreas-specific parameters, such as texture or thickness, were assessed comprehensively. In a recent meta-analysis^7^ of 8864 patients who had DP, smoking was shown to be a risk factor and diabetes a protective factor for CR-POPF after DP.

Preoperative pancreatic thickness (PT) has been associated with CR-POPF after DP. However, sample sizes were small, and no prediction model studies with PT have yet been published^8–14^. Assessment of intraoperative parameters is challenging in a minimally invasive setting; however, radiological parameters from preoperative CT images could provide the missing risk factors needed for successful risk stratification.

This study aimed to develop and externally validate a prediction model for CR-POPF after DP using radiological parameters from preoperative CT images, in addition to previously identified clinical risk factors.

## Methods

The TRIPOD statement^15^ was applied throughout the reporting of this study, and a checklist was completed (*supplementary material*).

This retrospective cohort study was approved by the institutional review boards of Helsinki University Hospital and Karolinska University Hospital. Data on consecutive patients undergoing DP from 1 January 2013 to 31 December 2021 at Helsinki University Hospital, Helsinki, Finland, were collected from electronic patient records for the development cohort. Consecutive patients undergoing DP from 1 January 2010 to 31 December 2020 at Karolinska University Hospital, Stockholm, Sweden, comprised the external validation cohort. Both centres are academic teaching hospitals that function as secondary and tertiary referral centres. Both open and minimally invasive DPs were included in this study. Patients with a history of pancreatic surgery were excluded. The pancreas was transected using a linear stapler or, if not feasible, a cold knife or scissors with resection line suturing. An intra-abdominal passive 24-Fr drain was always placed in both centres, and the decision regarding removal was based on the output and amylase concentration of the drain exudate during the early postoperative days. The drains were maintained until the output volume and amylase concentration were low. Perioperative somatostatin analogue treatment was used in both centres at the surgeon’s discretion. Pasireotide was used as a prophylactic in Helsinki, whereas octreotide was used as a treatment for high-output pancreatic fistulas in Stockholm.

### Missing data

**Fig. 1:**
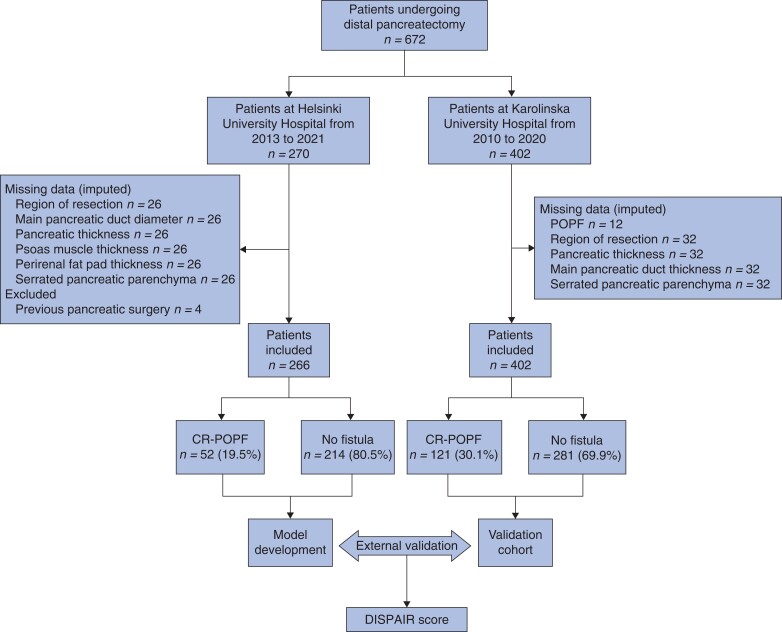
Study flow chart POPF, postoperative pancreatic fistula; CR, clinically relevant.

Missing data in both cohorts were assumed to be missing at random and imputed using multiple imputation (10 iterations, fully conditional specification), in which missing values were replaced with plausible values predicted from the associations between available data^16^. The following data were missing: 10 per cent of radiological parameters and smoking status in the development cohort, and 8 per cent of radiological parameters and 3 per cent of POPF status in the validation cohort (*Fig. 1*). Analyses were undertaken using pooled data from 10 imputation models.

### Outcome

CR-POPF was the primary outcome, and the 2016 International Study Group of Pancreatic Fistula definition was used^17^. Fistula occurrence and severity during the first 30 postoperative days were also recorded. Owing to the retrospective setting, the assessment of CR-POPF could not be blinded; however, its occurrence and severity were evaluated before the assessment of radiological parameters.

### Predictors

According to the literature, significant preoperative risk factors for CR-POPF after DP include young age, BMI, suspected high-risk pathology (non-malignant tumours), and smoking^6,7^. Diabetes was found to protect against CR-POPF^7^. The Charlson Co-morbidity Index^18^ was used to assess preoperative morbidity. Transection site was dichotomized between transection at the neck (at the portal/superior mesenteric vein) or body/tail of the pancreas.

**Fig. 2:**
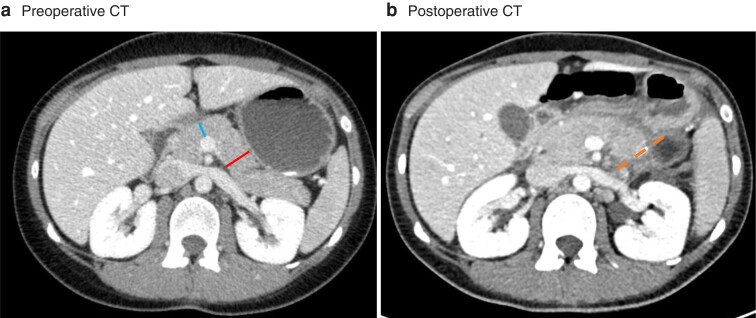
**Pancreatic thickness measurements a** Preoperative and **b** postoperative CT images. **a** Pancreatic thickness was measured at the transection site (23 mm, right [red] line), and at the neck of the pancreas (12 mm, left [blue] line). Main pancreatic duct diameter was measured at the same locations (not marked in this figure), and 1 mm was entered if not visible. The dashed (orange) line in **b** represents the transection site (body of pancreas in this patient).

Five preoperative radiological parameters were measured from the most recent preoperative CT images on a 5-mm axial section. The PT and main pancreatic duct diameter (MPDD) at the neck of the pancreas and at the site of transection were measured in millimetres as the width perpendicular to the pancreatic parenchyma (*Fig. 2*). The exact site of pancreas transection was assessed from postoperative CT images if available or the pathologist’s report using the margin from the tumour border as a guide. In addition, it was assessed whether or not the pancreatic parenchyma seemed lobular^19^. Perirenal fat pad thickness behind the left kidney, which served as a proxy for visceral fat, was measured in millimetres at the level of the renal hilum according to a published method (*Fig. S1*)^20,21^. Sarcopenia was assessed using a validated method^22^ by measuring the thickness of the right psoas major muscle in millimetres at the level of the third lumbar spine and dividing it by patient height (psoas muscle thickness per height, PMTH) (*Fig. S2*). Patients in the lowest PMTH quartile were considered sarcopenic. Assessors were blinded to patient outcomes; however, owing to the retrospective setting, postoperative CT images, operation texts, and pathologists’ reports were used to determine the transection site.

In addition to the main data collector, three independent observers assessed preoperative CT images from a randomly selected group of 50 patients from the development cohort, and interobserver agreement was assessed using the two-way random intraclass correlation coefficient (ICC) with absolute agreement. An ICC value of 0.75 or higher was considered satisfactory to prove the high reliability of measurements between observers. Variables with an ICC value below 0.75 were excluded from further analyses.

### Sample size

Considering the available sample size of the development cohort (266 patients) and an estimated outcome proportion of 25 per cent, the sample size was deemed to have adequate statistical power for the inclusion of eight candidate predictors for the logistic regression model when calculated as proposed by Riley *et al*.^23^.

### Statistical analysis

Continuous variables are reported as median (i.q.r.; range) and categorical variables as numbers with percentages. Differences in the distribution of variables between cohorts were assessed using the Mann–Whitney *U* test for continuous variables and Fisher’s exact test for categorical variables. Univariable analysis was undertaken using binary logistic regression with one independent variable to examine the associations between the study variables and CR-POPF. Fifteen preoperative variables were studied: age, sex, smoking, Charlson Co-Morbidity Index, BMI, diabetes, neoadjuvant therapy, sarcopenia, PT and MPDD at the neck and transection site, perirenal fat pad thickness, serrated/lobular pancreatic parenchyma, and transection site.

### Model development

Continuous variables were not categorized. Variables with the strongest univariable association were chosen to decrease the number of candidate predictors from 15 to 8. The linearity assumption for logistic regression was assessed by plotting. The model was created using binary logistic regression with eight candidate predictors using backward elimination based on the Akaike information criterion (AIC) to obtain the most parsimonious model^24^. Because prediction is about estimation, rather than hypothesis testing, two-sided *P* > 0.050 was not a criterion for omitting predictors from the model after AIC-based elimination^25^. Area under the receiver operating characteristic curve (AUROC) was used to assess discrimination.

### Model validation

Internal validation was performed using 1000 bootstrap resamples. The model was refitted in each of the bootstrap resamples and tested on the original sample to estimate the optimism in model performance. Optimism-adjusted AUROC was calculated as apparent AUROC (model discrimination in the original sample) minus optimism. To mitigate overfitting, a penalized maximum likelihood estimation with AIC was used for coefficient shrinkage (pentrace function, rms package; R)^26^. Penalized regression coefficients were used for the final model and calibration was assessed in the development cohort. External validation was done by analysing model discrimination and calibration in the Stockholm cohort. Calibration plots were drawn, and the slope, intercept, and Brier score were used to assess the calibration. Calibration in the large, which is measured by the model intercept in the calibration plot and reflects the average difference between the mean of observed outcomes and the mean of predicted outcomes, was used to assess the need for recalibration in the validation cohort. The model intercept was updated by adding a correction factor to better adjust for the mismatch in outcome proportion between cohorts, as described elsewhere^27^. Four risk groups were formed and tested for clinical applicability in the validation cohort.

In general, two-sided *P* < 0.050 was considered statistically significant. Data analysis was performed with SPSS^®^ version 27.0 for Macintosh^®^ software (IBM, Armonk, NY, USA) and R (R Core Team, Vienna, Austria). The rms package in R was used (R package version 6.2-0).

## Results

### Development cohort

Data from 672 patients undergoing DP from Finland and Sweden were included in the model development and validation cohorts respectively (*Fig. 1*). After excluding 4 patients with a history of pancreatic surgery, the final development cohort (Helsinki) comprised 266 patients, of whom CR-POPF occurred in 52 (19.5 per cent). The validation cohort (Stockholm) comprised 402 patients, 121 (30.1 per cent) of whom had CR-POPF. Basic clinical demographics and perioperative variables for both cohorts are shown in *Table 1*. There were statistically significant differences in CR-POPF occurrence, tumour histology, and Charlson Co-morbidity Index scores between the development and validation cohorts. A greater proportion of the pancreas was transected at the neck in the validation cohort (*Table 1*).

**Table 1:** Clinical demographics and perioperative details of patients undergoing distal pancreatectomy

	Development cohort, Helsinki (*n* = 266)	Validation cohort, Stockholm (*n* = 402)	*P*†
**Age (years), median (i.q.r.; range)**	64 (54–71; 18–89)	67 (56–73; 20–89)	0.073‡
**BMI (kg/m^2^), median (i.q.r.; range)**	26.23 (23.84–29.84; 17.21–44.62)	25.77 (22.70–29.33; 14.90–40.0)	0.068‡
**Charlson Co-morbidity Index score, median (i.q.r.; range)**	2 (1–4; 0–13)	1 (0–2; 0–10)	<0.001‡
**PT at pancreatic neck (mm), median (i.q.r.; range)**	12 (10–15; 5–23)	14 (12–17; 5–29)	<0.001‡
**PT at transection site (mm), median (i.q.r.; range)**	16 (13–20; 5–35)	15 (13–19; 5–32)	0.18‡
**MPDD at pancreatic neck (mm), median (i.q.r.; range)**	2.5 (1.5–3; 1–13)	2 (2–3, 1–13)	0.006‡
**MPDD at transection site (mm), median (i.q.r.; range)**	2 (1.5–3; 1–8)	2 (2–3; 1–13)	0.96‡
**Estimated blood loss (ml), median (i.q.r.; range)**	300 (100–800; 0–8800)	200 (100–500; 0–16 500)	<0.001‡
**Duration of initial hospital stay (days), median (i.q.r.; range)**	7 (5–9; 3–39)	7 (9–14; 3–133)	<0.001‡
**Men**	107 (40.2)	183 (45.5)	0.20
**Diabetes mellitus**	55 (20.7)	77 (19.2)	0.62
**Ever smoked**	92 (34.6)	n.a.	
**Neoadjuvant therapy**	32 (12.0)	12 (3.0)	<0.001
**Benign pathology**	91 (36.0)	185 (46.0)	0.012
Administration of somatostatin analogue*	147 (55.3)	108 (26.9)	<0.001
**Transection using stapler**	226 (85.0)	202 (50.2)	<0.001
**Serrated pancreatic parenchyma**	122 (45.9)	226 (56.2)	0.015
**Transection at pancreatic neck**	159 (59.8)	352 (87.6)	<0.001
**CR-POPF**	52 (19.5)	121 (30.1)	0.003

Values are *n* (%) unless otherwise indicated.

* Pasireotide used prophylactically at Helsinki University Hospital; octreotide used as a treatment for clinically relevant postoperative pancreatic fistula (CR-POPF) (grade B or C) at Karolinska University Hospital. PT, pancreatic thickness; MPDD, main pancreatic duct diameter; n.a., data not available. †Fisher’s exact test, except ‡Mann-Whitney *U* test.

### Interobserver agreement

Interobserver agreement between the four observers was satisfactory (ICC at least 0.75) for PT measurements at the transection site and neck, perirenal fat pad thickness, and psoas muscle thickness, indicating high reliability between observers (ICC 0.89, 0.89, 0.99, and 0.77 respectively). MPDD measurements at the transection site and neck, and assessments of whether the pancreatic parenchyma seemed serrated were not reliable (ICC 0.65, 0.69, and 0.60 respectively) (*Table 2*).

**Table 2:** Results of interoberver agreement between four independent observers assessing radiological parameters in 50 patients undergoing distal pancreatectomy

	Intraclass coefficient
**PT at transection site**	0.89 (0.83, 0.94)
**PT at pancreatic neck**	0.89 (0.83, 0.94)
**MPDD at transection site**	0.65 (0.46, 0.79)
**MPDD at pancreatic neck**	0.69 (0.51, 0.81)
**Psoas muscle thickness at third lumbar spine**	0.77 (0.43, 0.89)
**Perirenal fat pad thickness**	0.99 (0.98, 0.99)
**Assessment of pancreatic parenchyma as serrated/lobular or not**	0.60 (0.37, 0.76)

Values in parentheses are 95 per cent confidence intervals. An intraclass correlation coefficient value of 0.75 or higher was considered satisfactory to prove high reliability between measurements. PT, pancreatic thickness; MPDD, main pancreatic duct diameter.

### Model development and performance

According to the univariable analysis in the development cohort, PT, MPDD at the transection site, age, and history of diabetes were statistically significantly associated with CR-POPF (*Table 3*). The linearity of continuous predictors to the logit of CR-POPF was assessed by plotting, and no variables required transformation (*Fig. S3*).

**Table 3:** Univariable analysis of preoperative candidate predictors for pancreatic fistula in 266 patients undergoing distal pancreatectomy in development cohort (Helsinki University Hospital)

	OR	*P*
**Male sex**	0.82 (0.44, 1.54)	0.550
**Smoker**	1.60 (0.84, 3.04)	0.140
**Diabetes mellitus**	0.35 (0.13, 0.93)	0.034
**Neoadjuvant therapy**	0.55 (0.19, 1.65)	0.290
Sarcopenia*	0.76 (0.35, 1.62)	0.470
**Serrated pancreatic parenchyma**	1.64 (0.87, 3.10)	0.130
**Transection at pancreatic neck**	0.50 (0.27, 0.93)	0.027
**Age (years)**	0.98 (0.95, 0.99)†	0.016
**BMI (kg/m^2^)**	1.06 (0.99, 1.13)†	0.064
**Charlson Co-morbidity Index score**	0.93 (0.81, 1.06)†	0.280
**PT at pancreatic neck (mm)**	1.15 (1.05, 1.24)†	0.001
**PT at transection site (mm)**	1.51 (1.35, 1.68)†	<0.001
**MPDD at pancreatic neck (mm)**	0.97 (0.75, 1.25)†	0.790
**MPDD at transection site (mm)**	0.68 (0.47, 0.98)†	0.038
**Perirenal fat pad thickness (mm)**	0.99 (0.97, 1.02)†	0.640

Values in parentheses are 95 per cent confidence intervals.

* Defined as psoas muscle thickness per height in the lowest quartile.

† Per unit increase. PT; pancreatic thickness, MPDD; main pancreatic duct diameter.

Sarcopenia and perirenal fat pad thickness were excluded as they had a weak association with CR-POPF (*P* = 0.470 and *P* = 0.640 respectively) (*Table 3*). MPDD measurements and serrated pancreatic parenchyma were excluded because of low interobserver agreement (*Table 2*). Smoking was not available in the validation cohort and was therefore excluded. PT at the pancreatic neck was not as strongly associated with CR-POPF as PT at the transection site. For simplicity and to avoid multicollinearity, PT at the pancreatic neck was excluded from further model development. As a result, the final candidate predictors were age, BMI, PT at the transection site, neoadjuvant therapy, diabetes, sex, transection site, and Charlson Co-morbidity Index.

**Fig. 3:**
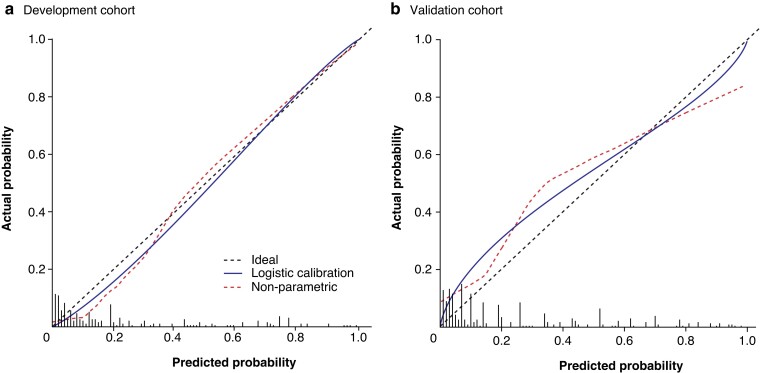
**Calibration plots for DISPAIR score development and validation cohorts a** Development cohort (Helsinki) and **b** validation cohort (Stockholm). Histograms represent the distribution of predicted risk. **a** AUROC 0.904, *R^2^* = 0.533, Brier score 0.084, intercept –0.109, slope 1.135; **b** AUROC 0.798, *R^2^* = 0.253, Brier score 0.162, intercept 0.192, slope 0.719.

In backward elimination, PT at the transection site, diabetes, sex, and transection site (pancreatic neck *versus* body/tail) remained in the model. Sex was excluded from the model because it has not been reported to be associated with CR-POPF after DP^6,7^ and had no effect in the validation cohort. The final model had an AUROC of 0.912 (95 per cent c.i. 0.864 to 0.959) and Nagelkerke *R*^2^ of 0.533 in the development cohort, and an AUROC of 0.904 (0.855 to 0.949) after internal validation, showing negligible model optimism (*Table 4*). In contrast, an otherwise similar model but with PT measured at the neck in all patients had an AUROC of 0.712 (0.625 to 0.773) in the development cohort. Smoking, which was not available in the validation cohort, did not contribute to the model’s performance in the development cohort as the AUROC with smoking included was 0.912 (0.863 to 0.960). Penalized regression coefficients were used in the final model (Table 4). The model was calibrated adequately (slope 1.135, calibration in the large −0.109) in the development cohort after penalization (*Fig. 3a*).

**Table 4:** Results of model development with binary logistic regression and internal validation of model

	Value	OR	β coefficient	*P*	Final model coefficients after penalization
**Apparent AUROC**	0.912 (0.864, 0.959)				
**Optimism-adjusted AUROC**	0.904 (0.855, 0.949)				
**Nagelkerke *R*^2^**	0.533				
**PT at transection site (mm)**		1.55 (1.38, 1.75)*	0.440*	<0.001	0.385*
**Transection at pancreatic neck**		2.00 (0.84, 4.80)	0.730	0.104	0.545
**Diabetes mellitus**		0.34 (0.10, 1.09)	−1.281	0.047	−1.116
**Intercept**			−9.894		−8.322

Values in parentheses are 95 per cent confidence intervals. Optimism-adjusted area under receiver operating characteristic curve (AUROC) calculated as apparent AUROC—optimism, which was acquired by bootstrapping.

* Per unit increase.

The possible confounding effects of stapler division, blood loss, and prophylactic somatostatin analogue use were assessed by entering them into a logistic regression analysis with the CR-POPF probabilities acquired from the final model in the development cohort. No significant confounding effect was found, with the respective ORs of 0.88 (95 per cent c.i. 0.22 to 3.54) (*P* = 0.862), 1.00 (0.99 to 1.01) per ml increase (*P* = 0.629), and 0.85 (0.35 to 2.06) (*P* = 0.723).

### External validation

External validation was performed in the validation cohort by assessing discrimination and calibration. The model had an AUROC of 0.798 (95 per cent c.i. 0.748 to 0.848) in the validation cohort (Stockholm). The calibration slope and Brier score were adequate (0.719 and 0.162 respectively); however, the calibration in the large (0.479) demonstrated that predictions were, on average, too low, probably owing to the higher incidence of CR-POPF in the validation cohort (30.1 *versus* 19.5 per cent; *P* = 0.002). A simple recalibration was performed by adjusting the regression model intercept by adding a correction factor, which resulted in a much better calibration in the large (0.192) while retaining satisfactory calibration in the development cohort (*Fig. 3*). The final CR-POPF probability equation for the proposed DISPAIR score is:

$$P=\;\frac{\exp\;(-8.322+0.384\;[\mathrm{Pancreatic}\;\mathrm{thickness}]+0.545\;[\mathrm{Transection}\;\mathrm{at}\;\mathrm{pancreatic}\;\mathrm{neck}]-1.116\;[\mathrm{Diabetes}])}{1+\exp(-8.322+0.384\;[\mathrm{Pancreatic}\mathrm{thickness}]+0.545\;[\mathrm{Transection}\;\mathrm{at}\;\mathrm{pancreatic}\;\mathrm{neck}]-1.116\;[\mathrm{Diabetes}])}$$

where *P* is the probability of CR-POPF, PT is measured at the transection site and entered as a continuous variable in millimetres, and transection at the pancreatic neck and diabetes as 1.0, if present.

Four risk groups were defined in the validation cohort for the clinical applicability of the DISPAIR score: low (below 5 per cent), moderate (at least 5 but below 30 per cent), high (at least 30 but below 75 per cent), and extreme (75 per cent or more). The incidence of CR-POPF in these groups was 8.7 per cent (11 of 126), 22.0 per cent (36 of 164), 63 per cent (57 of 91), and 81 per cent (17 of 21) respectively, in the validation cohort of 402 patients. Other postoperative outcomes stratified by risk group are shown in *Table S1*.

A guide on how to use the DISPAIR score and online calculator can be found at https://www.evidencio.com/models/show/2611.

### Sensitivity analyses

The performance of the DISPAIR score was assessed in different subpopulations to demonstrate adequate discrimination, regardless of the division technique or the administration of prophylactic somatostatin analogues (*Fig. S4*). The score performed similarly in all subgroups compared with its performance in the entire cohort of 668 patients, with an AUROC of 0.828 (95 per cent c.i. 0.790 to 0.866). The following AUROC values were acquired for the subpopulations: stump closure with stapler (428 patients), AUROC 0.822 (0.771 to 0.873); without stapler (240 patients), AUROC 0.837 (0.779 to 0.895); with administration of prophylactic somatostatin (147 patients), AUROC 0.891 (0.825 to 0.957); and without prophylactic somatostatin (521 patients), AUROC 0.820 (0.778 to 0.863).

## Discussion

The DISPAIR score is based on three preoperative variables showing high discrimination after external validation. The original fistula risk score for CR-POPF after pancreatoduodenectomy was developed in 2013^5^. Since its implementation, studies have been published validating and updating its model. Furthermore, updated risk scores, such as the alternative fistula risk score and the updated alternative fistula risk score, have been proposed^28,29^. According to a recent review^30^, the pooled AUROC values for these models in external validation studies published before June 2020 were 0.71, 0.70, and 0.72 respectively. Research on pancreatoduodenectomy has substantially benefited from the use of these risk scores. Perioperative risk stratification has allowed risk-adjusted analyses in clinical studies and directed the use of mitigation strategies, such as when to omit intra-abdominal drainage^31^ or the administration of perioperative somatostatin analogues^32^.

Despite clear demand, no similar risk scores for CR-POPF after DP have been formulated. In an international multicentre study of over 2000 patients, an attempt to develop such a prediction model demonstrated insufficient discrimination (AUROC 0.65)^6^. It was suggested that one reason for the poor performance of the models could have been some missing, unidentified risk factors. Although clinical demographics, such as previous morbidity, age, and BMI, are useful in risk stratification, CR-POPF is a process of the pancreatic stump, and pancreas-specific parameters are paramount for specifically capturing the risk profile of the pancreas. A few small-scale studies^8–14^ have identified PT at the transection site as a significant risk factor for CR-POPF after DP. Most of these studies had a small sample size (median 114) and used confusing categorization and combination of PT with other variables, such as Hounsfield units or stapler height, making meta-analysis challenging to perform. In the largest study to date assessing PT at the transection site, Sugimoto *et al*.^13^ showed PT to be an independent risk factor for CR-POPF (OR 1.19 per mm increase), which is in line with the present results.

The original fistula risk score and its derivatives are mainly based on the intraoperative assessment of pancreatic gland texture and MPDD. As most DPs are minimally invasive, reliable assessment of the pancreatic stump texture is challenging. Therefore, PT can function as a surrogate for gland texture in DP settings. In addition, it allows strong preoperative, as opposed to intraoperative, risk stratification. Measuring PT is more objective than gland texture assessment, because is it neither based on the subjective assessment made by the surgeon nor does it require dichotomization.

Although measuring PT at a fixed location, such as the pancreatic neck, could be more straightforward, it showed a weaker association with CR-POPF than PT at the transection site in both cohorts in the present study. Inclusion of transection site in the DISPAIR score allows the surgeon to assess different scenarios before operation and weigh up the risks of different approaches. In addition, measuring PT at the neck only might not fully capture the risk of a thick pancreas if resected at the body or tail. For example, in *Fig. 2*, measuring the thickness of the pancreatic neck would drastically underestimate the risk. Using the DISPAIR score could also be seen as a mitigation strategy, as the surgeon can choose where to transect the pancreas based on the predicted risk of different scenarios.

Transection at the pancreatic neck increased the risk of CR-POPF in this study. Although it may seem that transection at the neck carries a higher risk of CR-POPF, this risk is offset by the fact that the pancreas is usually thicker at the body/tail than at the neck. Mathematically, the effect of transection at the neck on the absolute probability of CR-POPF is approximately equal to an increase of 2 mm in PT in the DISPAIR score. However, the difference in PT between the transection sites was, on average, 4 mm in the development cohort and 3 mm in the validation cohort. Therefore, on average, the actual risk of CR-POPF is lower with transection at the neck, which is also demonstrated by the protective univariable association of transection at the pancreatic neck with CR-POPF in the development cohort (OR 0.50). However, in a scenario where the pancreas is uniform in thickness, transection at the body/tail region may be preferable in terms of CR-POPF mitigation. The effect of transection site on DP outcomes has been poorly studied, and there is no consensus on its effect or on the best site for transection^1,33,34^. However, the present findings add to the scarce body of evidence that, in certain situations, the transection site could affect DP outcomes.

A history of diabetes has been associated with a lower CR-POPF incidence^7^ and is also a DISPAIR score predictor. This effect of diabetes on CR-POPF might be due to some alterations in pancreatic histology, and is not explained solely by PT. It was also observed that MPDD cannot be reliably measured from preoperative CT images despite its possible association with CR-POPF. Including MPDD in a model would add significant observer-dependent variability to the predictions. In contrast, PT measurements varied little between the observers. To the authors’ knowledge, the present study is the first to demonstrate this.

The DISPAIR score has many potential applications, owing to its preoperative setting. Although incorporating intraoperative factors in the model could have provided additional information, the benefit of planning and preoperative decision-making would have been lost. Even though it might be impossible to know the exact transection site before surgery, the DISPAIR score allows the surgeon to assess the risk of CR-POPF at different transection sites, enabling an informed decision to be made during the operation. Because of the lack of a validated risk stratification system, no reliable risk-adjusted analyses in RCTs of DP and CR-POPF have been conducted. Preoperative risk stratification allows guided decision-making, such as the administration of somatostatin analogues or whether intra-abdominal drainage is needed. This could potentially increase the cost-effectiveness of DP, as patients in need of mitigation strategies could be better identified from those who do not benefit from them. The DISPAIR score could enable a risk-adjusted comparison of surgical outcomes between operators and lead to a better assessment of surgeon-specific learning.

This study had some limitations. Data on both cohorts were collected retrospectively, and were prone to misclassification and recall bias. The sample size of the development cohort was inadequate to include all the preoperative variables in model development, and some variables (such as sarcopenia and perirenal fat pad thickness) had to be excluded from the final model development. Therefore, some potentially contributing risk factors were omitted from the model. Information on smoking, which has been reported to be associated with CR-POPF, was not available for the validation cohort and this variable was excluded from model development. However, smoking did not significantly contribute to model performance in the development cohort. Because the incidence of CR-POPF varies between centres and the incidence in the development cohort was at the lower end, the model intercept was adjusted to provide slightly higher absolute risk estimates on average (calibration in the large). In theory, this recalibration warrants a new external validation; in practice, no huge difference would be expected, as other calibration measures were not adjusted for. In addition, there were other statistically significant differences between the development and validation cohorts, such as in Charlson Co-morbidity Index scores, tumour histology, and PT at the neck. Although this might be one reason for a significant drop in the model’s discrimination between cohorts (0.90 *versus* 0.80), the AUROC on external validation can still be considered very satisfactory and has clinical applicability as previously used fistula risk scores for pancreatoduodenectomy have externally validated AUROCs of approximately 0.70. In addition, it can be argued that the model shows good transportability, because it discriminates well in a cohort with significantly different baseline characteristics. However, although the authors do not expect the effect of PT on CR-POPF to vary significantly between countries, DISPAIR in essence has only been validated in the Nordic population and validation in different populations is encouraged. As the population undergoing DP is heterogeneous in terms of surgical protocol (including division techniques and somatostatin analogues), there is a risk of selection bias. The present sensitivity analyses demonstrated that DISPAIR discriminates well, regardless of the protocol used, and so can be used as a fistula risk estimation tool for the whole population undergoing DP.

One strength of this study was the use of robust statistical methods^15,35–38^. Missing data were imputed^16^, interobserver agreement of radiological measurements was assessed, continuous variables were not categorized^39^, and geographical external validation was performed with an adequate sample size (over 100 events)^40^.

## Supplementary Material

znac266_Supplementary_Data
