# Supplementary material for: Development and external validation of DISPAIR fistula risk score for clinically relevant postoperative pancreatic fistula risk after distal pancreatectomy
Source: Br J Surg. 2022 Aug 19;109(11):1131–9. doi: 10.1093/bjs/znac266 (PMC10364701; doi:10.1093/bjs/znac266)
Supplement: znac266_Supplementary_Data [file znac266_supplementary_data.docx]

**Supplemental material**

**Table of Contents**

**TRIPOD Checklist.**

**Table S1. Postoperative outcomes of 668 patients undergoing distal pancreatectomy, stratified by four risk groups for pancreatic fistula derived with the DISPAIR-score.**

**Figure S1. Measuring perirenal fat pad thickness from preoperative imaging prior to distal pancreatectomy.**

**Figure S2. Measuring psoas major muscle thickness from preoperative imaging prior to distal pancreatectomy.**

**Figure S3. Graphical representation of continuous variables with logit of clinically relevant postoperiative fistula. Development cohort, 266 patients undergoing distal pancreatectomy.**

**Figure S4. Results of sensitivity analyses, the performance of the DISPAIR-score in four different subpopulations measured with calibration plots.**

**TRIPOD Checklist.**

| **Section/Topic** | **Item** |  | **Checklist Item** | **Page** |
| --- | --- | --- | --- | --- |
| **Title and abstract** | | | | |
| Title | 1 | D;V | Identify the study as developing and/or validating a multivariable prediction model, the target population, and the outcome to be predicted. | 1 |
| Abstract | 2 | D;V | Provide a summary of objectives, study design, setting, participants, sample size, predictors, outcome, statistical analysis, results, and conclusions. | 2-4 |
| **Introduction** | | | | |
| Background and objectives | 3a | D;V | Explain the medical context (including whether diagnostic or prognostic) and rationale for developing or validating the multivariable prediction model, including references to existing models. | 5 |
|  | 3b | D;V | Specify the objectives, including whether the study describes the development or validation of the model or both. | 6 |
| **Methods** | | | | |
| Source of data | 4a | D;V | Describe the study design or source of data (e.g., randomized trial, cohort, or registry data), separately for the development and validation data sets, if applicable. | 6 |
|  | 4b | D;V | Specify the key study dates, including start of accrual; end of accrual; and, if applicable, end of follow-up. | 6 |
| Participants | 5a | D;V | Specify key elements of the study setting (e.g., primary care, secondary care, general population) including number and location of centres. | 6 |
|  | 5b | D;V | Describe eligibility criteria for participants. | 6 |
|  | 5c | D;V | Give details of treatments received, if relevant. | 6 |
| Outcome | 6a | D;V | Clearly define the outcome that is predicted by the prediction model, including how and when assessed. | 7 |
|  | 6b | D;V | Report any actions to blind assessment of the outcome to be predicted. | 7 |
| Predictors | 7a | D;V | Clearly define all predictors used in developing or validating the multivariable prediction model, including how and when they were measured. | 7-8 |
|  | 7b | D;V | Report any actions to blind assessment of predictors for the outcome and other predictors. | 8 |
| Sample size | 8 | D;V | Explain how the study size was arrived at. | 8 |
| Missing data | 9 | D;V | Describe how missing data were handled (e.g., complete-case analysis, single imputation, multiple imputation) with details of any imputation method. | 6-7 |
| Statistical analysis methods | 10a | D | Describe how predictors were handled in the analyses. | 8-9 |
|  | 10b | D | Specify type of model, all model-building procedures (including any predictor selection), and method for internal validation. | 8-10 |
|  | 10c | V | For validation, describe how the predictions were calculated. | 9 |
|  | 10d | D;V | Specify all measures used to assess model performance and, if relevant, to compare multiple models. | 9 |
|  | 10e | V | Describe any model updating (e.g., recalibration) arising from the validation, if done. | 9 |
| Risk groups | 11 | D;V | Provide details on how risk groups were created, if done. | 9,12 |
| Development vs. validation | 12 | V | For validation, identify any differences from the development data in setting, eligibility criteria, outcome, and predictors. | 6 |
| **Results** | | | | |
| Participants | 13a | D;V | Describe the flow of participants through the study, including the number of participants with and without the outcome and, if applicable, a summary of the follow-up time. A diagram may be helpful. | 10, Figure 2 |
|  | 13b | D;V | Describe the characteristics of the participants (basic demographics, clinical features, available predictors), including the number of participants with missing data for predictors and outcome. | 6-7,10,Table 1 |
|  | 13c | V | For validation, show a comparison with the development data of the distribution of important variables (demographics, predictors and outcome). | Table 1 |
| Model development | 14a | D | Specify the number of participants and outcome events in each analysis. | 10, Figure 2 |
|  | 14b | D | If done, report the unadjusted association between each candidate predictor and outcome. | eTable 2 |
| Model specification | 15a | D | Present the full prediction model to allow predictions for individuals (i.e., all regression coefficients, and model intercept or baseline survival at a given time point). | 12, Table 2 |
|  | 15b | D | Explain how to the use the prediction model. | 12 |
| Model performance | 16 | D;V | Report performance measures (with CIs) for the prediction model. | 11-12 |
| Model-updating | 17 | V | If done, report the results from any model updating (i.e., model specification, model performance). | 12 |
| **Discussion** | | | | |
| Limitations | 18 | D;V | Discuss any limitations of the study (such as nonrepresentative sample, few events per predictor, missing data). | 15 |
| Interpretation | 19a | V | For validation, discuss the results with reference to performance in the development data, and any other validation data. | 13 |
|  | 19b | D;V | Give an overall interpretation of the results, considering objectives, limitations, results from similar studies, and other relevant evidence. | 12-14 |
| Implications | 20 | D;V | Discuss the potential clinical use of the model and implications for future research. | 15 |
| **Other information** | | | | |
| Supplementary information | 21 | D;V | Provide information about the availability of supplementary resources, such as study protocol, Web calculator, and data sets. | 16 |
| Funding | 22 | D;V | Give the source of funding and the role of the funders for the present study. | 1 |

**Table S1. Postoperative outcomes of 668 patients undergoing distal pancreatectomy, stratified by four risk groups for pancreatic fistula derived with the DISPAIR-score.**

| **Outcome** | **Low risk (<5%)**  **n=227** | **Moderate risk (5-30%)**  **n=248** | **High risk (30-75%)**  **n=130** | **Extreme risk (>75%)**  **n=63** | **p-value** |
| --- | --- | --- | --- | --- | --- |
| **Reoperation, n=39, n(%)** | 7 (3.1) | 13 (5.2) | 9 (6.9) | 10 (15.9) | 0.002 |
| **CR-POPF, n=173, n(%)** | 13 (5.7) | 41 (16.5) | 72 (55.4) | 47 (74.6) | <0.001 |
| **Clavien Dindo IIIa or worse, n=182, n(%)** | 31 (13.7) | 59 (23.8) | 56 (43.1) | 36 (57.1) | <0.001 |
| **CR-DGE, n=23, n(%)** | 9 (4.0) | 6 (2.4) | 3 (2.3) | 5 (7.9) | 0.094 |
| **90 day mortality, n=12, n(%)** | 2 (0.9) | 6 (2.4) | 3 (2.3) | 1 (1.6) | 0.608 |
| **CR-PPH, n=33, n(%)** | 7 (3.1) | 12 (4.8) | 7 (5.4) | 7 (11.1) | 0.235 |

CR-POPF; clinically relevant postoperative pancreatic fistula, CR-DGE; clinically relevant delayed gastric emptying, CR-PPH; clinically relevant postpancreatectomy hemorrhage. P-value derived with Chi-squared test.

**Figure S1. Measuring perirenal fat pad thickness from preoperative imaging prior to distal pancreatectomy.**

**
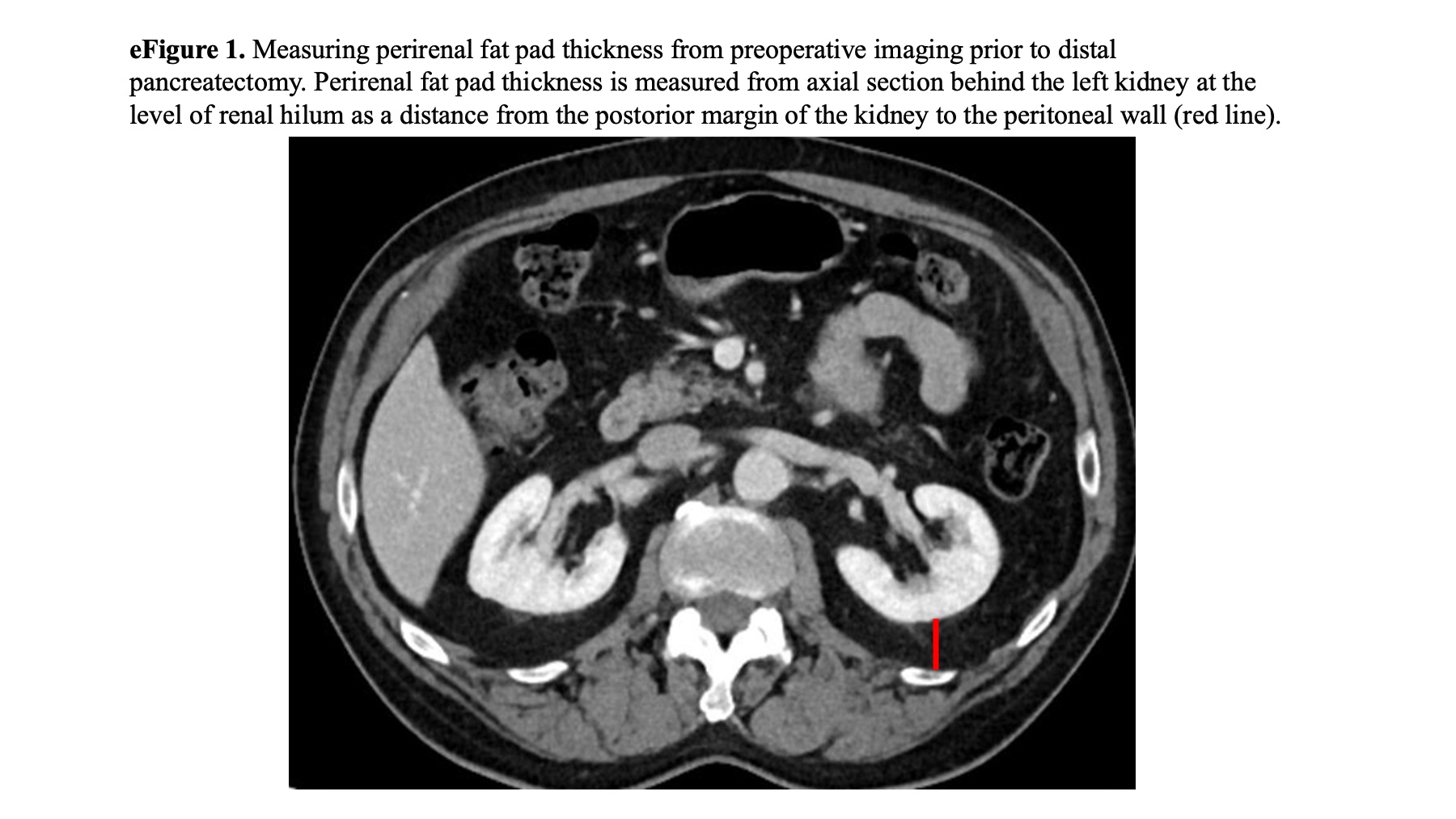
**

Perirenal fat pad thickness is measured from axial section behind the left kidney at the level of renal hilum as a distance from the postorior margin of the kidney to the peritoneal wall (red line).

**Figure S2. Measuring psoas major muscle thickness from preoperative imaging prior to distal pancreatectomy.**

**
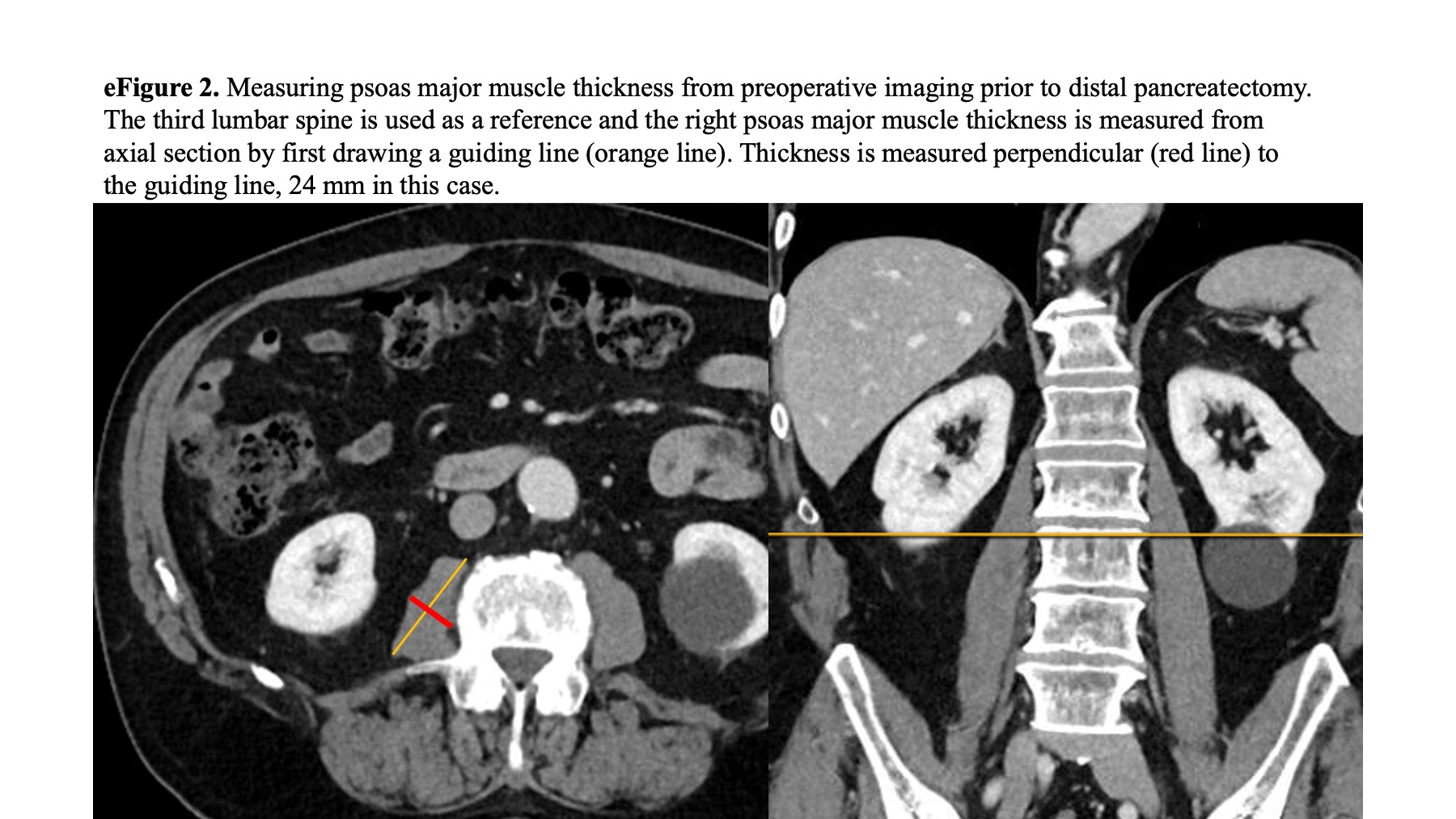
**

The third lumbar spine is used as a reference and the right psoas major muscle thickness is measured from axial section by first drawing a guiding line (orange line). Thickness is measured perpendicular (red line) to the guiding line, 24 mm in this case.

**Figure S3. Graphical representation of continuous variables with logit of clinically relevant postoperiative fistula. Development cohort, 266 patients undergoing distal pancreatectomy.**

Logit of postoperative pancreatic fistula on X-axis, continous variable on Y-axis. MPDD; main pancreatic duct diameter, PT; pancreatic thickness.

**Figure S4. Results of sensitivity analyses, the performance of the DISPAIR-score in four different subpopulations measured with calibration plots.**

Triangles represent risk deciles.
